# Impacts of spaceflight experience on human brain structure

**DOI:** 10.1038/s41598-023-33331-8

**Published:** 2023-06-08

**Authors:** Heather R. McGregor, Kathleen E. Hupfeld, Ofer Pasternak, Nichole E. Beltran, Yiri E. De Dios, Jacob J. Bloomberg, Scott J. Wood, Ajitkumar P. Mulavara, Roy F. Riascos, Patricia A. Reuter-Lorenz, Rachael D. Seidler

**Affiliations:** 1 Department of Applied Physiology and Kinesiology, University of Florida, Gainesville, FL USA; 2 Russell H Morgan Department of Radiology and Radiological Science, The Johns Hopkins University School of Medicine, Baltimore, USA; 3 Departments of Psychiatry and Radiology, Brigham and Women’s Hospital, Harvard Medical School, Boston, MA USA; 4 KBR, Houston, TX USA; 5 NASA Johnson Space Center, Houston, TX USA; 6 Retired, NASA Johnson Space Center, Houston, TX USA; 7 University of Texas Health Science Center at Houston, Houston, TX USA; 8 Department of Psychology, University of Michigan, Ann Arbor, MI USA; 9 Norman Fixel Institute for Neurological Diseases, University of Florida, Gainesville, FL USA

**Keywords:** Neuroscience, Neurology

## Abstract

Spaceflight induces widespread changes in human brain morphology. It is unclear if these brain changes differ with varying mission duration or spaceflight experience history (i.e., novice or experienced, number of prior missions, time between missions). Here we addressed this issue by quantifying regional voxelwise changes in brain gray matter volume, white matter microstructure, extracellular free water (FW) distribution, and ventricular volume from pre- to post-flight in a sample of 30 astronauts. We found that longer missions were associated with greater expansion of the right lateral and third ventricles, with the majority of expansion occurring during the first 6 months in space then appearing to taper off for longer missions. Longer inter-mission intervals were associated with greater expansion of the ventricles following flight; crew with less than 3 years of time to recover between successive flights showed little to no enlargement of the lateral and third ventricles. These findings demonstrate that ventricle expansion continues with spaceflight with increasing mission duration, and inter-mission intervals less than 3 years may not allow sufficient time for the ventricles to fully recover their compensatory capacity. These findings illustrate some potential plateaus in and boundaries of human brain changes with spaceflight.

## Introduction

Spaceflight imposes multiple hazards on the human body including increased radiation, microgravity exposure, and social isolation and confinement in a closed environment, among other factors^1^. A number of studies have now reported that spaceflight alters human brain morphology^2–13^. Spaceflight induces an upward shift of the brain within the skull^10,14^, resulting in cortical crowding and narrowing of the sulci at the top of the brain^12,14^. This is reflected as widespread gray matter volume (GMv) increases at the top of the brain and GMv decreases around the base of the brain^11,12,14^. Post-flight GM shifts are accompanied by displacement of intracranial fluid^3,12,15^ including extracellular free water (FW) such as cerebrospinal fluid. Following spaceflight, there are decreases in intracranial fluid volume at the top of the brain and increases around the base of the brain^3,10,12,13,15^. Ventricular expansion also occurs with spaceflight, with reported average volume increases ranging from 11 to 25%^2–4,6,8,9,12,14–16^. However, astronauts vary considerably in their current and past spaceflight experience. Mission durations typically range from 2 weeks to 1 year, and some astronauts are novices while others are experienced (with varying numbers of prior flights and inter-mission intervals). It is not known if or how these individual differences in prior flight experience are associated with spaceflight-induced structural brain changes and intracranial fluid shifts. Gaining insight into experience-dependent structural brain changes with spaceflight is crucial with multi-year human missions to Mars on the horizon. Determining whether brain changes continue throughout prolonged microgravity exposure or plateau at some point during flight will help us to better understand the nature and mechanisms of these changes.

Previous studies have leveraged the flight duration differences between short-duration (2 week) space shuttle missions and long-duration (6 months or more) International Space Station (ISS) missions to study the impact of spaceflight duration on brain changes. Six-month missions result in larger GMv shifts than 2-week missions^11,14^, and year-long ISS missions induce even greater GMv increases within sensorimotor cortical regions compared to 6-month missions^12^. These findings suggest that longer time in microgravity results in greater cortical crowding at the apex of the brain. Roberts and colleagues also found greater ventricular volume expansion following 6-month flights than 2-week flights^4,14^. Spaceflight results in brain white matter (WM) microstructure changes within tracts subserving vestibular function^10^, visual function^8^, visuospatial processing^10^, and sensorimotor control^10,13^. Longer duration missions result in smaller microstructural changes within the cerebellar WM compared to shorter missions^10^. Although it seems counterintuitive that there would be a greater change in this structure for shorter missions, this may reflect an early, adaptive structural change in-flight that gradually returns to baseline over time. Current models of training-associated neuroplasticity suggest a similar pattern, with gray matter expansion occurring during periods of performance improvement and then returning to pre-training levels as performance change begins to plateau^17,18^.

Our group has also reported associations between FW shifts and individual differences in previous spaceflight experience^10^. Astronauts who had completed a greater number of previous missions showed FW decreases in the anterior, medial portion of the brain whereas less experienced astronauts showed FW increases in this region^10^. This pattern was irrespective of the flight duration and cumulative number of days in space, indicating that the number of gravitational transitions experienced (as opposed to the amount of time spent in space) has an important effect on the brain’s FW distribution^10^. It is possible that repeated adaptation to multiple gravitational transitions may affect the gross morphology of the brain. There is also recent evidence of holdover effects from previous flights with novice astronauts—but not experienced astronauts—showing increases in perivascular spaces within the white matter^19^. Time between successive missions may also impact spaceflight-induced brain changes. Ventricular enlargement persists after spaceflight, showing only partial recovery in the following 6–12 months^3,6,12,13^. We recently reported that astronauts with less recovery time between missions had larger ventricles pre-flight (even after correcting for age effects), and they showed smaller ventricular volume increases with subsequent missions^12^. These findings suggest that crewmembers with larger ventricles pre-flight (whether due to older age or prior spaceflight experience) have less available room or compliance for ventricular expansion with spaceflight.

Here we examined whether and how spaceflight-induced brain changes interact with individual differences in current and previous spaceflight experience including: the duration of a mission, whether a crewmember was novice or experienced, their number of previous missions, and time elapsed since a previous mission. We leveraged MRI data from a sample of 30 astronauts who varied along the following flight experience dimensions: mission duration (approx. 2 weeks to 1 year), previous spaceflight experience (0 to 3 previous missions), and time since previous flight (approx. 1 to 9 years). We conducted whole-brain voxelwise analyses assessing associations between these individual differences and changes in GMv, FW fractional volume, FW-corrected WM microstructure, and ventricular volume. We used a voxelwise analysis to localize structural changes associated with spaceflight experience to specific brain areas; this is particularly relevant given the functional specialization of different regions across the brain. In light of previous work, we hypothesized that longer mission durations would induce greater GMv and FW shifts, greater ventricular expansion, and smaller WM microstructure changes. We hypothesized that greater previous flight experience and smaller inter-mission intervals would induce smaller brain structure changes and FW shifts. The latter finding would suggest reduced compliance/elasticity following a prior flight.

## Results

### Spaceflight induces gray matter shifts, free water redistribution, and ventricular enlargement

We assessed group-level pre- to post-flight changes in GMv, ventricular volume, FW fractional volume, and FW-corrected WM diffusion indices. Analyses were adjusted for astronaut age at launch, sex, mission duration, and time elapsed between landing and the post-flight MRI scan. Two-tailed t-test results were thresholded at p < 0.05 with FWE correction.

We found statistically significant GMv shifts following spaceflight with apparent GMv increases at the top of the brain and GMv decreases around the base (Fig. S1), replicating previous results of previous spaceflight^3,11,12,14^ and bed rest analog studies^20^. We observed statistically reliable enlargement of the lateral and third ventricles following spaceflight (Fig. S2; Table 2) as observed in previous studies^3,4,6,8,9,12,14,16^ We observed no statistically reliable group level changes in FW-corrected WM diffusion indices from pre- to post-flight. Also replicating our previous work^3,12–14^, spaceflight resulted in widespread decreases in FW fractional volume around the vertex of the brain and FW fractional volume increases around the lower temporal and frontal lobes (Fig. S3).

### Longer missions induce greater fluid shifts

We tested for pre- to post-flight changes in GMv, ventricular volume, FW fractional volume, or WM diffusion indices that scaled with the crewmember’s actual current mission duration, in days. Here, we use “current mission” to refer to a crewmember’s most recent mission for which we have pre- and post-flight MRI scans. Analyses were adjusted for astronaut age, sex, and time elapsed between landing and the post-flight MRI scan. Two-tailed t test results were thresholded at p < 0.05 with FWE correction.

**Figure 1:**
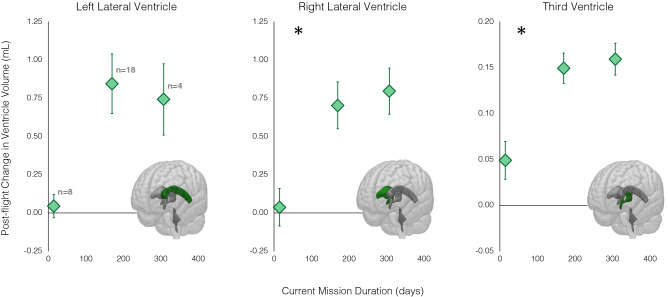
Pre- to post-flight ventricular volume changes associated with current mission duration. 2-week-long missions resulted in smaller increases (or in some instances decreases) in ventricle volume compared to missions lasting 6 months or longer. The duration of the current mission, in days, was used in the statistical models. To protect crewmember privacy, the plots show average values of subgroups of astronauts who completed 2-week-long (n-8), 6-month-long (n = 18) or 1-year-long missions (n = 4). Individuals gave permission for data presentation when group sizes are smaller than five. Subgroup sample sizes are indicated in gray on the leftmost plot. The lower right corner of each plot shows the ventricles overlaid on a rendered MNI standard space brain template, with the referent ventricle shown in green. Asterisks indicate results that survived the Benjamini–Hochberg FDR correction. Error bars represent standard error of the mean.

As shown in Table 2, mission duration was associated with pre- to post-flight increases in left lateral ventricle volume (β = 0.0039, p = 0.018), right lateral ventricle volume (β = 0.0037, p = 0.006), and third ventricle volume (β = 0.00056, p = 0.0004) though only the results for the right lateral and third ventricle survived FDR correction using the Benjamini–Hochberg method (see “Methods”). As shown in Fig. 1, 2-week-long missions (n = 8) resulted in smaller increases (or in some instances decreases) in right lateral and third ventricle volume compared to missions lasting 6 months (n = 18) or longer (n = 4).

**Figure 2:**
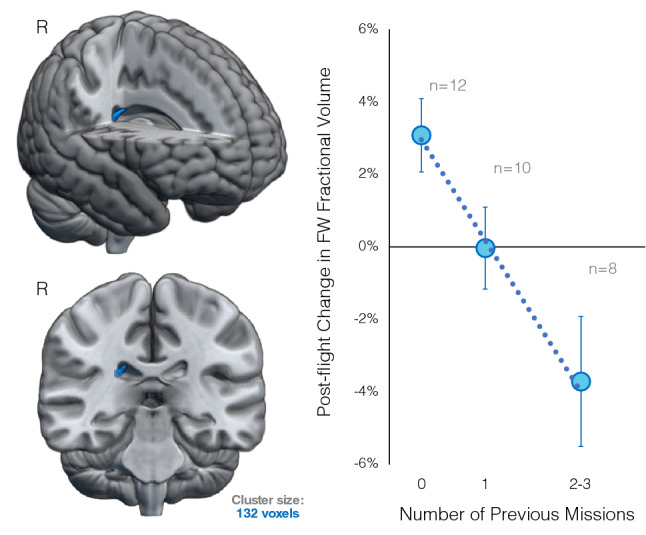
Pre- to post-flight FW volume changes associated with previous number of missions. Cluster located at the outer wall of the right lateral ventricle overlaid on a rendered MNI standard space template (left). The scatterplot shows that having completed a greater number of previous missions was associated with greater post-flight FW volume decreases within this cluster. The dotted line indicates the linear fit of the individual data points. Circular markers indicate the average of subgroups, the size of each subsample is indicated in gray. The 2 and 3 previous missions subgroups have been combined to protect crewmember privacy for data visualization; they were not combined for the statistical analyses. Error bars represent standard error. Results are FWE corrected at p < 0.05, two-tailed. R indicates the right hemisphere. FW, free water.

We found no statistically reliable associations between current mission duration and pre- to post-flight changes in GMv, FW shifts, or WM microstructure.

### No differences in structural brain changes between novice and experienced astronauts

We tested for differences in post-flight GMv, ventricular volume, FW fractional volume, or WM diffusion index changes between novice (n = 12) and experienced flyers (n = 18). We categorized astronauts based on whether or not they had any previous spaceflight experience. “Novice” astronauts were those who had no previous spaceflight experience whereas “experienced” flyers were those who had previously completed one or more previous missions. Analyses were adjusted for astronaut age, sex, mission duration, and time elapsed between landing and the post-flight MRI scan. Two-tailed t-test results were thresholded at p < 0.05 with FWE correction.

Whole-brain analyses revealed no statistically reliable associations between post-flight changes in GMv, ventricular volume (see Table 2), FW fractional volume, or WM microstructure within the tissue compartment (i.e., fractional anisotropy (FA_t_), axial diffusivity (AD_t_), and radial diffusivity (RD_t_)) and whether a crewmember was a novice or experienced flyer.

### Greater prior flight history associated with decreases in FW following spaceflight

We then assessed whether the *extent* of previous spaceflight experience was associated with spaceflight-induced brain changes. We examined pre- to post-flight changes in GMv, ventricular volume, FW fractional volume, or WM diffusion indices that were associated with the number of prior missions completed. Analyses were adjusted for astronaut age, sex, mission duration, and time elapsed between landing and the post-flight MRI scan. Two-tailed t test results were thresholded at p < 0.05 with FWE correction.

There were no statistically reliable associations between the number of previous missions completed and post-flight GMv shifts or ventricular volume changes.

Voxelwise FW analyses revealed a cluster at the edge of the right lateral ventricle in which post-flight changes in FW fractional volume were associated with the number of previous missions crewmembers had completed. As shown in Fig. 2, novice (n = 12) astronauts exhibited FW fractional volume increases within this region following spaceflight. In contrast, crewmembers who had completed multiple previous missions (n = 8) tended to show FW decreases within this region. Within each subgroup, (i.e., number of previous missions), FW changes observed in crewmembers with altered DWI acquisition parameters were within range (i.e., Z scores <  ± 1.3) of FW changes observed in those crewmembers with consistent acquisition parameters.

Analyses yielded no statistically reliable associations between the number of previous missions completed and FW-corrected WM diffusion indices examined.

### Greater ventricular expansion for astronauts with longer inter-mission intervals

We tested whether post-flight changes in GMv, ventricular volume, FW fractional volume, or WM diffusion indices scaled with the time interval between successive missions. Analyses were adjusted for astronaut age, sex, and time elapsed between landing and the post-flight MRI scan. Two-tailed t test results were thresholded at p < 0.05 with FWE correction.

We found no statistically reliable associations between the time elapsed since the previous mission and post-flight GMv shifts.

**Figure 3:**
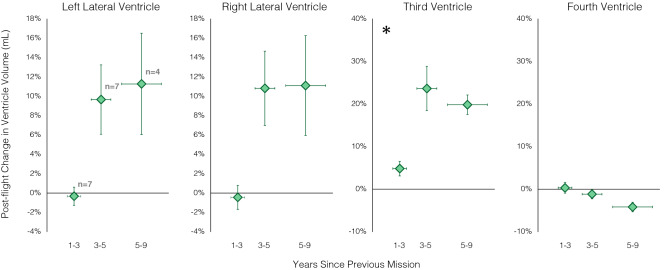
Pre- to post-flight ventricle volume changes associated with inter-mission intervals. Pre- to post-flight ventricle volume changes of the third ventricle were significantly associated with the number of years since the previous mission’s end. Actual inter-mission intervals were used in the statistical models. Crewmembers have been subgrouped for data visualization only to protect their privacy. The x axis shows exclusive ranges (i.e., encompassing inter-mission intervals up to, but excluding the upper bound). Markers indicate subgroup averages with subgroup sample sizes indicated in gray in the leftmost plot. Error bars represent standard error. Asterisks indicate results that survived the Benjamini–Hochberg FDR correction.

Among the experienced astronauts, the number of years elapsed since the previous mission was significantly associated with post-flight volume changes for all four ventricles (see Fig. 3). Longer time between successive missions was associated with greater increases in left lateral (β = 0.23668, p = 0.0481), right lateral (β = 0.21517, p = 0.0314), and third ventricle (β = 0.0305, p = 0.0081) volumes following spaceflight. The fourth ventricle showed the opposite pattern with longer inter-mission delays being associated with greater volumetric decreases following spaceflight (β = -0.0120, p = 0.0488). However, only the association between third ventricle volume changes and years since the previous mission survived FDR correction using the Benjamini–Hochberg correction^20^. For experienced crewmembers, shorter inter-mission intervals (typically < 3 years) were associated with smaller increases (or in one astronaut a decrease) in the volume of the third ventricle. Longer inter-mission intervals were associated with larger expansion of the third ventricle following spaceflight (Table 2, Fig. 3). It is possible that these associations were driven more so by the duration of the previous mission or the previous number of missions completed than the inter-mission interval per se. For example, previously completing a 2-week or 6-month mission may have different carryover effects on the current mission, impacting a crewmember’s pre-flight ventricle size or brain compliance. To address this issue, we performed follow-up analyses on our significant tests by adding these covariates to our linear mixed models: the duration of the previous mission (actual number of days), the number of previous missions, or both. In all cases, the results of the model remained statistically significant regardless of variability in the duration of the previous flight and/or the number of previous flights completed.

There were no statistically reliable associations between time since the previous mission and changes in FW volume or FW-corrected WM diffusion indices.

## Discussion

Here we showed that longer duration spaceflight resulted in greater ventricular enlargement, which appeared to taper off after 6 months in space. Longer recovery time between subsequent missions was associated with greater post-flight ventricular expansion.

### Associations with mission duration

Longer duration missions were associated with greater enlargement of the right lateral ventricle and third ventricle following spaceflight, though the rate of ventricle volume expansion appears to slow for missions over 6 months in duration. Both the right lateral and third ventricles showed greater expansion for missions lasting longer than 2 weeks. Crewmembers who completed 6-month and 1-year-long missions showed a similar degree of expansion of these ventricles following flight, providing preliminary evidence that changes begin to taper off during 6-month-long missions. This pattern suggests that the majority of ventricle changes in flight occur during one’s first 6 months in space. However, there is considerable variability between crewmembers in terms of ventricle changes during year-long missions. Moreover, this preliminary result should be interpreted with caution as it is based on a small sample (n = 4); data from additional 1-year crewmembers are needed to shed additional light on ventricle expansion patterns.

Ventricles enlarge over time as part of the normal aging process^22^; however, the rate of ventricular expansion during spaceflight exceeds that seen with aging^5,6,12^. Several groups have reported no changes in global GM and WM volume following spaceflight^2,4,9,11^ (though see^6^). This suggests that post-flight ventricular enlargement is not a consequence of brain atrophy with normal aging, but instead arises from CSF changes. While the precise mechanism underlying this phenomenon remains unclear, disruptions in the CSF dynamics have been hypothesized as an underlying cause. Headward fluid shifts in microgravity may impair CSF outflow and cause cerebral venous congestion^23,24^. Cortical crowding and compression at the top of the brain (resulting from the upward brain shift^14^) may impede CSF flow through the subarachnoid space along the superior cortical surface and/or impair CSF resorption into the superior sagittal sinus via the arachnoid granulations^16^. Ventricular enlargement may thus reflect a compensatory response to accommodate increased intracranial CSF volume^2^.

### Associations with previous flight experience

Novice crewmembers showed FW fractional volume increases at the edge of the right lateral ventricle following spaceflight while those with experience of 2 or more previous missions showed FW fractional volume decreases within this region. Using a subset of the prospective astronaut data, we previously showed that crewmembers who had completed more previous missions showed smaller enlargement of the right lateral ventricle^12^. This may be due to cumulating carryover effects from previous flights. That is, the brains of more experienced crewmembers may be less compliant because they have previously undergone ventricular expansion during multiple flights and slow recovery following spaceflight^2,6,12^. As a result, the ventricles of more experienced crewmembers may not be able to expand, reducing their efficacy as an overflow zone for fluid shifts and reduced CSF resorption during missions^2,5,16^. This would be especially the case if the brain does not fully recover between flights and crewmembers begin a subsequent flight with enlarged ventricles^12^, which may have been the case for one of the 1-year crewmembers noted above. In contrast, the brains of novice or less experienced crewmembers may have greater compliance, allowing their ventricles to expand in space to adapt for in-flight cephalad fluid shifts.

It is worth noting that prior flight experience may increase with crewmember age, however we adjusted for individual differences in crewmember age in all of our analyses (in addition to sex, current mission duration, etc.). Therefore, variance in crewmember age does not account for the observed individual differences in structural brain changes. This finding is consistent with previous reports showing that the rate of ventricular volume expansion during long duration spaceflight exceeds the rate of ventricle expansion with normal healthy aging on Earth^5,12^. This suggests that the aging process or age differences are not the primary factor driving the observed post-flight brain changes. Our results suggest that repeated adaptation to changing gravity conditions experienced across multiple previous flights alters FW fractional volume. There is already an interest in behavioral responses to gravitational transitions, as crewmembers typically show aftereffects of adaptation to the previous context. For example, transient aftereffects of adaptation of vestibular processing to microgravity are evident when individuals return to Earth as mobility and balance declines. We suggest that the brain changes we observed here in relation to the number of previous missions stem from differing effects. That is, they do not occur in sensory brain regions thought to play a role in multisensory reweighting following spaceflight^21^, but rather occur adjacent to the lateral ventricles. This implies that the effects are more associated with structural adaptations occurring in response to the intracranial fluid and brain position shifts that occur with spaceflight^3,11,14,22^.

We found no statistically reliable differences in spaceflight-induced brain changes between astronauts when categorizing them on a binary basis as novice or experienced. Instead, differential brain changes emerged when we factored in the number of prior flights the experienced astronauts had completed. This finding suggests that the brain is impacted by the cumulative effects across multiple flights and perhaps separate bouts of adaptation to microgravity and the spaceflight environment.

### Associations with inter-mission intervals

Among the experienced astronauts, crewmembers who had less than 3 years of time to recover following their previous mission showed little to no enlargement of the lateral and third ventricles following the current mission. In contrast, those crewmembers who had 3 years or longer to recover following their previous mission showed ventricular expansion following the current mission. Ventricular expansion during spaceflight may be compensatory, allowing the brain to accommodate fluid shifts towards the head that occur in microgravity^2^. Ventricular expansion resolves slowly post-flight^2,12^, with crewmembers showing an average of ~ 55–64% recovery towards pre-flight levels 6–7 months following a 6-month ISS mission^2,12^. It is currently unclear how long it takes the ventricles to fully recover post-flight or to what extent they recover. Incomplete ventricular recovery between flights may negatively impact this compensatory mechanism during a subsequent flight. For example, if the ventricles are already enlarged pre-flight, they may be less compliant and/or have less space to expand during in-flight, reaching their maximum capacity sooner and storing less excess CSF during the following mission. Qualitatively, in the current study, most of the crewmembers with inter-mission intervals of 3 years or longer exhibited post-flight lateral and third ventricle expansion (see Fig. 3). This suggests that crewmembers may require a 3-year interval between successive missions for post-flight ventricular recovery and regaining structural compensatory capacity. Using a subset of the prospective data, our group previously reported that less time between successive missions was associated with larger pre-flight lateral ventricles (an effect that was not attributable to age)^12^. Analysis of pre-flight ventricle volumes and inter-mission intervals using the larger dataset in the current study did not replicate this effect, resulting in mixed evidence. We previously reported that less time between successive missions was associated with smaller ventricular volume increases with flight^12^. We observed a similar effect here, suggesting incomplete recovery between successive flights. Future multi-year longitudinal studies could shed more light on post-flight ventricular recovery.

### Limitations

A limitation of this work includes differing MRI scan parameters for a subset of diffusion-weighted MRI images for the retrospective subjects (see “Methods”), with some scan parameters differing between pre- and post-flight scans, though significant FW changes observed in crewmembers with altered DWI acquisition parameters were within range (i.e., Z scores <  ± 1.3) of FW changes observed in those crewmembers with consistent acquisition parameters. Similar to previous studies^2–5,10,11^, post-flight MRI scans occurred an average of approximately 6 days following landing (range: 1–20 days). It is possible that some spaceflight-induced brain changes recovered prior to the post-flight brain scan or were impacted by readaptation to Earth’s gravity. Although we adjusted for the time delay between landing and the post-flight MRI scan session in all analyses, this time delay was longer for astronauts in the retrospective dataset. Prospective studies could also apply more advanced dMRI acquisition approaches such as multi-shell and multi-band which would make the model estimations more accurate and will shorten the length of the acquisition.

## Conclusion

Here we reported how current and previous spaceflight experience relates to spaceflight-induced brain structural changes. Longer duration missions induced greater ventricular expansion, though the majority of ventricle volume changes appear to occur within the first 6 months in space, with the rate of ventricle change slowing beyond 6 months. A greater number of prior missions was associated with reductions in FW volume along the border of the right lateral ventricle. Longer inter-mission intervals were associated with greater post-flight ventricular expansion. Collectively, this work suggests that longer missions, multiple flights, and shorter inter-mission recovery time induce greater intracranial fluid changes. With human spaceflight becoming more frequent and lengthy, these findings provide important insight into how spaceflight experience, both current and previous, impacts the brain and suggest potential guidelines for future mission planning. Moreover, the findings suggest that neuroplasticity changes resulting from adaptation to microgravity may not be dependent on previous spaceflight experience. However, we have a small sample of longer duration flyers, and the timeline of pre- and post-flight data collections are not optimized to match what is currently known about human neuroplasticity^22–24^.

## Methods

### Participants

T1-weighted and diffusion-weighted MRI (dMRI) scans collected from a total of 30 astronauts were included in this study. Data from 15 of the astronauts were collected as part of a prospective study conducted between 2014 and 2020^25^. Astronauts in the prospective group completed a long-duration mission to the ISS lasting approximately 6 (n = 13) or 12 months (n = 2). Data from the remaining 15 astronauts were obtained from the NASA Lifetime Surveillance of Astronaut Health Repository. We obtained MRI scans from 28 crewmembers from this database. Data from 13 of these astronauts were excluded due to missing pre-flight T1 or dMRI scans (n = 10), incomplete brain coverage (n = 1), insufficient number of diffusion-weighted volumes acquired (n = 1), or participation in our prospective study (n = 1). Astronauts in the retrospective group completed a short-duration mission lasting approximately 2 weeks (n = 8) or a long-duration ISS mission lasting approximately 6 months (n = 5) or 1 year (n = 2). Crewmember demographic information is presented in Table 1. In this paper, we use “current mission” to refer to a crewmember’s most recent mission for which we have accompanying pre- and post-flight MRI data.

**Table 1:** Astronaut demographics.

	Short-duration spaceflight (n = 8)	Long-duration spaceflight (n = 22)
Sex	7 males, 1 female	16 males, 6 females
Mean age at launch (SD)	48 (2.3) years	46.8 (5.7) years
Current mission duration (SD)	14.5 (1.6) days	195 (60) days
Day of post-flight scan (SD)	12.0 (6.3) days	4.1 (1.8) days
Novice/experienced	8 experienced	12 novices, 10 experienced
Experienced astronaut demographics		
Previous number of missions (SD)	1.5 (0.76) missions	1.7 (0.82) missions
Previous flight experience (SD)	79.6 (81.1) days	119.3 (143.6) days
Number of years since previous mission end (SD)	2.3 (0.6) years	5.3 (1.4) years

SD, standard deviation. Experienced refers to having completed at least 1 previous spaceflight mission prior to enrollment in the current study.

This study was approved by the institutional review boards at the University of Michigan, University of Florida, and the NASA Johnson Space Center. All methods were performed in accordance with the relevant guidelines and regulations. Crewmembers provided written informed consent prior to participating in the prospective study. This manuscript and all figures underwent attributability review by NASA’s Lifetime Surveillance of Astronaut Health team and, where required, individual crewmember consent was obtained for data presentation.

### MRI acquisition

T1-weighted anatomical scans and diffusion-weighted MRI (dMRI) scans were acquired pre- and post-flight. All neuroimaging data were acquired using the same 3 T Siemens Magnetom Verio MRI scanner located at University of Texas Medical Branch at Victory Lake in Houston, TX.

T1-weighted anatomical images for all astronauts were collected using a magnetization-prepared rapid gradient-echo (MPRAGE) sequence with the following parameters: TR = 1900 ms, TE = 2.32 ms, flip angle = 9°, inversion time = 900 ms, FOV = 250 × 250 mm, 176 sagittal slices of 0.9 mm thickness, matrix = 512 × 512, voxel size = 0.488 × 0.488 × 0.9 mm, acquisition time = 4.5 min.dMRI scans were acquired using a 2D single-shot spin-echo prepared echo-planar imaging sequence. Prospective dMRI scans were acquired using the following parameters: TR = 11,300 ms, TE = 95 ms, flip angle = 90°, FOV = 250 × 250 mm, matrix size = 128 × 128, 40 axial slices of 2 mm thickness (no gap), voxel size = 1.95 × 1.95 × 2 mm, acquisition time = 11.5 min. Thirty non-collinear gradient directions with diffusion weighting of b = 1000 s/mm^2^ were sampled twice. A volume with no diffusion weighting (b = 0 s/mm^2^) was acquired at the start of each sampling stream. Retrospective dMRI scans were acquired using the following parameters: TR = 5800 ms, TE = 95 ms, flip angle = 90°, FOV = 250 × 250 mm, matrix size = 128 × 128, 40 axial slices of 3.9 mm thickness (no gap), voxel size = 1.95 × 1.95 × 3.9 mm. Twenty non-collinear gradient directions with diffusion weighting of b = 1000 s/mm^2^ were sampled three times. At the beginning of each sampling stream, a volume with no diffusion weighting (b = 0 s/mm^2^) was acquired. Voxel dimensions were altered for 9 retrospective crewmembers resulting in a voxel size of 1.8 × 1.8 × 3.9 mm for 9 preflight and 1 post-flight scans. Repetition times were also altered for 4 retrospective crewmembers as follows: 4 pre-flight scans (TR = 5846, 5500, 5900, 5600 ms) and 1 post-flight scan (TR = 5302 ms).

### T1-weighted image preprocessing

All T1-weighted images underwent identical preprocessing using the Computational Anatomy Toolbox^26^ (CAT12.6 v.1450) for Statistical Parametric Mapping^27^ version 12 (SPM12 v.7219) implemented using MATLAB R2016a, version 9.0.

After visual inspection, native space T1 images were skull stripped using CAT12. We additionally segmented each native space T1 image into gray matter (GM), white matter (WM), and cerebrospinal fluid (CSF) segments. GM segments were used in subsequent analyses of gray matter volume (GMv).

CAT12 automatically estimated the volumes, in mL, of the left lateral, right lateral, third, and fourth ventricles using the Neuromorphometrics volume-based atlas map included in SPM12. Ventricular volume analyses were performed in native space. CAT12 additionally provided estimates of each astronaut’s total intracranial volume. Pre-flight total intracranial volumes were included in GMv and ventricular volume analyses detailed below.

### dMRI preprocessing

All dMRI scans underwent identical preprocessing and analyses using FMRIB Software Library (FSL) version 6.0.1^28^, and a custom FW algorithm^29^ implemented in MATLAB R2018b.

Raw dMRI scans were visually inspected for scan artifacts and excessive head movement. We used FSL’s preprocessing tool, *eddy,* to correct for eddy current distortions and inter-volume head movement. Diffusion-weighted volumes (b = 1000 s/mm^2^) were registered to the average of the b = 0 volumes in the run. Rotations applied to each volume during motion correction were also applied to corresponding diffusion gradient directions. A volume was deemed an outlier if the root mean square voxel displacement was greater than 1 mm relative to the previous volume. Outlier volumes and those with artifacts were removed from the eddy corrected image and from b-value and b-vector matrices. Preprocessed dMRI data were then skull stripped using FSL’s brain extraction tool, *bet*.

FW maps and FW-corrected diffusion indices were computed by fitting a bi-tensor model at each voxel. This model, detailed in^29^, has been used in several studies using different DWI acquisition parameters (e.g.,^10,15,30,31^). Briefly, the model consists of FW and tissue compartments: The FW component first models water molecules that are free to diffuse as isotropic diffusion with a diffusion coefficient of water at body temperature (3 × 10^−3^ mm^2^/s). For estimating the tissue compartment, a diffusion tensor is used to model the diffusivity of water molecules within tissue. These components yield FW maps and FW-corrected diffusion indices, respectively. FW maps reflect the fractional volume of the FW compartment within each voxel; these values range from 0 to 1, where 1 indicates that a voxel is filled entirely by freely-diffusing water molecules. The tissue compartment yields the following diffusion index maps: FW-corrected fractional anisotropy (FA_t_), FW-corrected axial diffusivity (AD_t_), and FW-corrected radial diffusivity (RD_t_) that are calculated from the diffusion tensor.

### Image normalization

As in our previous work^10–12^, we used a multi-step process to improve within-subject registration of our longitudinal images^32^. Registration was performed using Advanced normalization tools (ANTs) version 1.9.17^33,34^. As described below, this multi-step approach involves registering a subject’s native space images to standard space via subject-specific templates.

We first generated subject-specific T1 templates. Subject-specific T1 templates were created by averaging the subject’s native space skull-stripped pre-flight and post-flight T1 images using antsMultivariateTemplateConstruction.sh. In this way, the template was not biased towards either time point. Each subject-specific T1 template was then warped to a 1 mm-resolution MNI standard space T1 template using rigid, affine, and Symmetric Normalization (SyN) transformations using ANTs. Using the method, we also created subject-specific FA templates using subject’s FA images that were not FW-corrected. Native space FA maps were eroded by 1 voxel to remove noise around the outer edge of the volume. Subject-specific FA templates were normalized to an FA template in MNI space as above.

The above steps generated transformations for registering each native space image (T1 or FA) to a subject-specific template, and from a subject-specific template to MNI space. We concatenated these transformations into flow fields to minimize the number of interpolations performed during normalization. A separate flow field was generated for each native space T1 image and FA map. Since FA, FW, FA_t_, AD_t_, and RD_t_ maps from a given session were derived from the same dMRI image, the same flow field was applied to transform each into MNI standard space. These steps yielded MNI-normalized GM segments, FW maps, and diffusion index maps with a resolution of 1 mm^3^.

### GM modulation

As in our previous work^11^, flow fields that were applied to GM segments were additionally used to estimate tissue expansion and shrinkage following spaceflight. Each flow field was inputted to ANTs’ CreateJacobianDeterminantImage.sh function to obtain the Jacobian determinant image. The Jacobian determinant encodes local expansion and shrinkage for each voxel within the image. We then multiplied each MNI-normalized GM segment by its corresponding Jacobian determinant image to produce modulated GM segments in standard space for each subject and session. Modulation preserves the amount of GM present in the untransformed image.

### Masking

Voxelwise FW analyses were performed within a whole-brain mask including the ventricles and CSF around the brain parenchyma. Analyses of WM diffusion indices were confined to the WM. A binary WM mask was created by thresholding all MNI-normalized FA maps (≥ 0.2) to identify WM, and included only those voxels in which WM was present in more than half of the sample^10^.

### Smoothing

Modulated GM segments were smoothed using an 8 mm (full width at half maximum) FWHM Gaussian kernel to increase signal-to-noise ratio. MNI-normalized FW maps and FW-corrected diffusion indices were each smoothed using a 5 mm FWHM Gaussian kernel.

### Group level analyses

#### Statistical models

Our first model tested for significant brain changes from pre- to post-flight averaged across all astronauts. We then used 4 models to examine associations between pre- to post-flight brain changes and the following crewmember individual differences: current mission duration, whether crewmembers were experienced or novice, previous number of missions (excluding the current mission), and years since previous mission end (for experienced crewmembers only).

Models adjusted for individual differences in age at the time of launch, sex, current mission duration, and the number of days between landing and the post-flight MRI scan (except for models in which one of these covariates was the predictor of interest). GMv and ventricular volume analyses also adjusted for pre-flight total intracranial volume.

#### Whole-brain voxelwise analyses

For each astronaut, we calculated FW difference images reflecting pre- to post-flight changes in FW fractional volume. We then concatenated the astronauts’ FW difference images into a single 4-dimensional image. This procedure was repeated individually for each image type.

We performed one-sample t tests on the GMv, FW, FA_t_, RD_t_, AD_t_ difference images using *randomise*, FSL’s tool for nonparametric permutation-based inference^35^. All tests were performed using 15,000 random permutations with threshold-free cluster enhancement^36^. Correction for multiple comparisons was implemented using familywise error (FWE) correction (*p* < 0.05, two-tailed).

Since the DWI acquisition parameters were altered in a subset of crewmembers, we performed additional follow-up analyses for all significant FW and ADt results in which we compared changes observed in the crewmembers with altered DWI acquisition parameters against those with consistent acquisition parameters. Within each subgroup, (i.e., same mission duration or previous missions), we computed the Z score for each crewmember with altered DWI acquisition relative to the crewmembers with consistent acquisition parameters. In this way, we could assess whether the changes observed in crewmembers with consistent acquisition parameters were within range of observed in crewmembers with consistent acquisition parameters.

#### Ventricular volume analyses

We analyzed pre- to post-flight changes in ventricular volumes using linear mixed models using restricted maximum likelihood (REML). Analyses were implemented in R version 3.3.3 using the nlme package^37^. Our linear mixed models modeled random effects corresponding to subject-specific intercepts and slopes. Time was modeled as a categorical variable (i.e., pre-flight or post-flight session). We performed a total of 20 ventricular volume analyses (as shown in Table 2), with 5 analyses performed for each of our 4 ventricle ROIs); we used the Benjamini–Hochberg procedure to maintain our FDR at p < 0.05^20^. Results of our linear mixed model analyses were submitted to Benjamini-Hochberg’s FDR procedure with *α*FDR = 0.05. Results were considered statistically significant at a p < 0.0025 (i.e., (1/20) × 0.05))^20^. This approach was chosen as a compromise between controlling type I errors and type II error rates in this highly unique dataset. The Benjamini–Hochberg FDR procedure helped to control FDR inflation (allowing examination of spaceflight-induced changes for each individual ventricle) while being less stringent than Bonferroni correction.

**Table 2:** Pre- to post-flight ventricular volume changes.

Model	Ventricle							
	Left Lateral		Right Lateral		Third		Fourth	
	β	p	β	p	β	p	β	p
Session (pre-flight, post-flight)	0.61455	**0.0001**	0.5100	**0.0001**	0.1189	**0.0000**	− 0.00669	0.5702
Current mission duration	0.0033	0.0210	0.0032	**0.0061**	0.00049	**0.0004**	− 0.000016	0.8978
Novice vs. experienced	0.0343	0.9056	0.0709	0.7682	0.0182	0.5417	0.03344	0.1634
Previous number of missions	− 0.1221	0.3943	− 0.0881	0.4611	− 0.0124	0.400	− 0.0241	0.039
Years since previous mission end	0.2367	0.0481	0.2151	0.0314	0.0305	**0.0081**	− 0.0120	0.0488

Beta and p values listed are those corresponding to the variable of interest for each statistical model shown in the leftmost column. Statistically reliable results (p < 0.05) are underlined. Results that additionally survived the Benjamini–Hochberg FDR correction are bolded.

## Supplementary Information

Supplementary Information.

## Data Availability

MRI files for this study will be placed in the NASA LSDA data repository upon study completion (https://lsda.jsc.nasa.gov/explore/lsdahome/).
